# Effect of medication review and cognitive behaviour treatment by community pharmacists of patients discharged from the hospital on drug related problems and compliance: design of a randomized controlled trial

**DOI:** 10.1186/1471-2458-10-133

**Published:** 2010-03-15

**Authors:** Abeer Ahmad, Jacqueline Hugtenburg, Laura MC Welschen, Jacqueline M Dekker, Giel Nijpels

**Affiliations:** 1 Department of Clinical Pharmacology and Pharmacy and the EMGO Institute for Health and Care Research, VU University Medical Center, Amsterdam, The Netherlands; 2 Department of General Practice and the EMGO Institute for Health and Care Research, VU University Medical Center, Amsterdam, The Netherlands; 3 Department of Epidemiology and Biostatistics and the EMGO Institute for Health and Care Research, VU University Medical Center, Amsterdam, The Netherlands

## Abstract

**Background:**

Drug related problems (DRPs) are common among elderly patients who are discharged from the hospital and are using several drugs for their chronic diseases. Examples of drug related problems are contra-indications, interactions, adverse drug reactions and inefficacy of treatment. Causes of these problems include prescription errors and non-compliance with treatment. The aim of this study is to examine the effect of *medication review *and *cognitive behaviour therapy *of discharged patients by community pharmacists to minimize the occurrence of drug related problems.

**Methods/Design:**

A randomized controlled trial will be performed. Community pharmacists will be randomized into a control group and an intervention group. 342 Patients, aged over 60 years, discharged from general and academic hospitals, using five or more prescription drugs for their chronic disease will be asked by their pharmacy to participate in the study.

Patients randomized to the control group will receive usual care according to the Dutch Pharmacy Standard. The medication of patients randomised to the intervention group will be reviewed by the community pharmacist with use of the national guidelines for the treatment of diseases, when patients are discharged from the hospital. The Pharmaceutical Care network Europe Registration form will be used to record drug related problems. Trained pharmacy technicians will counsel patients at home at baseline and at 1,3,6,9 and 12 months, using Cognitive Behaviour Treatment according to the Theory of Planned Behaviour. The patient's attitude towards medication and patient's adherence will be subject of the cognitive behaviour treatment. The counselling methods that will be used are *motivational interviewing *and *problem solving treatment*. Patients adherence towards drug use will be determined with use of the Medication Adherence Report Scale Questionnaire. There will be a follow-up of 12 months.

The two primary outcome measures are the difference in occurrence of DRPs between intervention and control group and adherence with drug use. Secondary endpoints are attitude towards drug use, incidence of Re-hospitalisations related to medicines, functional status of the patient, quality of life and the cost-effectiveness of this intervention.

**Discussion:**

Combining both medication review and Cognitive Behaviour Treatment may decrease DRPs and may result in more compliance with drug use among patients discharged from the hospital and using 5 or more chronic drugs.

**Trial registration:**

Dutch Trial Register NTR1194

## Background

Drug related problems (DRPs) are events or circumstances involving drug therapy that actually or potentially interfere with desired health outcomes [1]. Examples of DRPs are contra-indications, interactions, adverse drug reactions (ADR) and inefficacy of treatment. Causes for these problems can be prescription errors, non-compliance with treatment and the specific effects of drugs in patients. Factors that increase the risk of DRPs are polypharmacy, co-morbidity, aging, non-adherence and lack of coordination between different treating physicians.

An increased number of prescribed drugs (polypharmacy) strongly increase the risk of DRP. Runciman found a correlation between increases in medication use and rates of adverse drug reactions associated with hospitalization [2]. Another recent review of studies of the effect of polypharmacy on the health state of elderly people has shown that multiple drug use is a strong predictor of hospitalisations, nursing home placement, death, hypoglycaemia, fractures, impaired mobility, pneumonia and malnutrition [3]. Furthermore, Leendert et al suggest that elderly people have a higher risk of hospitalisation caused by DRP, especially if they use have 4 or more co-morbidity [4].

Elderly people >75 year seem at higher risk for hospitalisation caused by DRP [2]. A study conducted in the Netherlands examined the occurrence of hospitalisations that were related to medication. This study showed 12.793 acute hospital admissions per year of which 714 admissions were medication related and 332 of these admissions were preventable. They calculated that 19.000 hospital admissions per year were related to medication and were preventable [4]. Specific risk factors were the number of prescribing physicians [5] and the number of diagnoses [6], the number and combination of several (inappropriate) drugs [6,7] and the use of inappropriate drugs [8-11].

Non-adherence is another risk factor for the occurrence of DRP. Assuming that drugs have been prescribed correctly, non-compliance may substantially affect the efficacy of treatment or even enhance the risk of side effects [12-14]. In the Dutch population 50% of the patients were shown to discontinue the use of chronic medication within one year after initiation. Discontinuation of chronic medication depended on the type of chronic medication and occurred frequently among patients who were using antihypertensive medication, cholesterol lowering drugs, anti-osteoporoses drugs, anti-rheumatic medications and antidepressants [15].

Hospitalisation can also be the cause of DRP. Hospitalisation and subsequent discharge are associated with discontinuity of care [16]. At the point of discharge the use of certain drugs may have been discontinued and the doses of others changed while new medication may have been added. In the Netherlands, pharmacists use a computerized system to detect drug problems among their patients. This system fails to detect all drug problems and does not provide information on problems with medication use, which elderly patients may experience. In the present study, pharmacists will perform a medication review on elderly patients discharged from the hospital. This medication review is a structured, critical examination of patient's medicines with the objective of reaching an agreement with the patient about treatment, optimising the impact of medicines, minimising the number of medication-related problems and reducing waste [17].

A systematic review conducted by Royal et al. concluded that there was evidence showing that pharmacist-initiated interventions including a medication review component are effective in reducing hospital admissions by 36%. Several studies have shown that cognitive behaviour treatment can be useful in improving medication adherence among patients [18,19]. Patients may benefit of changes in attitude to medication, resulting in increased compliance with drug use.

The present randomized controlled study aims to improve pharmacotherapy by means of combining two effective strategies including medication review and cognitive behaviour treatment. To our knowledge, the effect of combining both methods to minimize drug related problems and improve compliance among elderly patients discharged from the hospital has not been studied previously.

### Theoretical framework the cognitive behavioural approach

**Figure 1:**
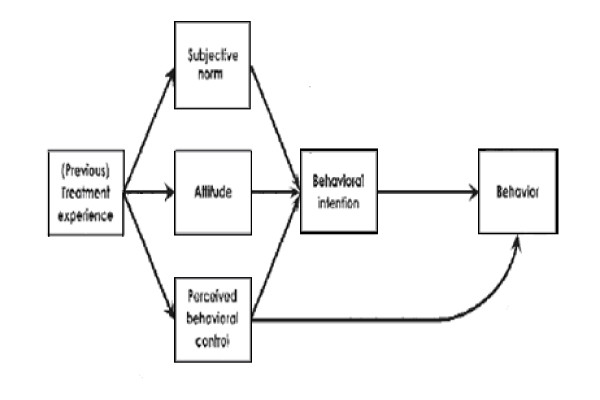
**Model for Medication Adherence: theory of planned behaviour**.

Studies have shown that a change in patients attitude to medications may enhance adherence to medications [16-20]. Behavioural interventions may increase this effect [16-20]. A behavioural model of medication adherence is shown in figure 1. This model is based on the Theory of Planned Behaviour and provides insight into the factors that may determine adherence behaviour [18,19]. Patients gain 'treatment experience' once they are exposed to a medical regimen. To enable patients to adequately make informed choices about their behaviour and to motivate themselves to execute the behaviour correctly and at the right time, patients must possess a basic level of understanding about their illness. Health care professionals must communicate this information in understandable and concrete language, tailored to the needs of each specific patient [19]. The most important concept used in the adherence model is the intention of the patient to adhere to the medical recommendations. The Theory of Planned Behaviour states that the intention to adhere is determined by the subjective norm, the attitude and the perceived behavioural control [18]. The subjective norm is what a person thinks other people believe (s) he should do (e.g. physicians or partners) and the motivation to comply with these normative references. The subjective norm of a patient can be changed through either changing the patients perception of the norm, or through changing the patients motivation to comply with this norm.

The attitude reflects salient beliefs about the perceived outcomes of these behaviours (e.g. adherence leads to better health than non-adherence) and the evaluations of these outcomes (e.g. good health is essential for living longer). It is important for the patient to realise that the benefit of adhering to the medical regimen outweigh the costs. It is therefore important that doctors and community pharmacists explain to the patient what the benefits are of medication adherence. The patient will then be able to make his own informed choice about his medicines and outweigh the costs of this behaviour.

The last component is the perceived behavioural control (PBC). The PBC has two components: self-efficacy dealing with the perceived ease or difficulty of performing behaviour, such as feeling confident to always take medication correctly in a private setting and controllability the extent to which the behaviour is up to the person. If a person has a demanding job it is sometimes difficult to remember intake or have some privacy. To optimize someone's PBC, it is useful to separate behaviour in small and simple steps and to facilitate the behaviour where possible (for example: use dose organizers) and plan the execution of the behaviour in a setting in which the patient feels confident about correctly performing the behaviour on a daily basis.

The outcome of the attitude, subjective norm and perceived behaviour control is expressed in a behavioural intention. This intention leads to accepting or refusing adherence to the treatment as prescribed [19].

### Medication review

Medication review is an intervention that can be used to prevent the occurrence of DRP. Medication review requires access to the patients notes, full record of prescriptions and non-drug care and results from laboratory tests etc. The medicines used by the patient will be reviewed in the context of the patients condition and the perspective of the patient. In this process the patient is involved as a full partner. This means listening to the patient's views and beliefs about their medicines, reaching an honest understanding of their medicine taking behaviour and taking full account of their preferences in any decisions about treatment. Possible detected DRPs are communicated with the prescriber, in order to find a solution for these problems. Any changes made will then be communicated with the patient.

### Cognitive behaviour treatment

To increase concordance with drug use, according to our theoretical framework, we have developed a combined intervention with motivational interviewing (MI) and Problem Solving Treatment (PST). MI is used to increase patient's motivation towards concordance with medication prescription [21]. When patients experience barriers with medication use, PST will be used in order to give patients the tools to overcome these barriers [22].

#### Motivational interviewing

Motivational interviewing is a client-oriented counselling method that is shown to be effective in improving health behaviour [21]. Increasing the intrinsic motivation of patients can then lead to a positive behavioural change. During the counselling sessions the therapist does not take an expert role but rather a role as a partner. MI includes 5 counselling techniques aimed at helping patients resolve ambivalence about health behaviour: (1) expressing empathy; (2) developing discrepancy; (3) avoiding argument (4) rolling with resistance; and (5) supporting self-efficacy [21]. When patients are motivated to change their health behaviour, the next step is to increase their self-management towards this behaviour by using PST.

#### Problem solving treatment

This intervention increases the ability of patients to solve their problems in a structured way and improve their confidence in dealing with future problems. The treatment aims to give patients the tools to overcome barriers in order to stimulate structural healthy behaviour. During the treatment session there is an active collaboration between the patient and the pharmacist, in which the patient takes an active role in the planning of his treatment [23]. PST can be considered as a series of 7 stages [22].

1. Explanation of the intervention and its rationale

2. Definition and breaking down of the problem

3. Establishing achievable goals for the problem resolution. Achieve goals are SMART goals: Specific, Measurable, Achievable, Relevant, Timed

4. Generating multiple possible solutions

5. Evaluating and choosing the solution

6. Implementing the preferred solution

7. Evaluating the outcome

### Objectives of this study

The objective of this study is to investigate the effects of a multifaceted intervention, existing of medication review and a CBT intervention for medication adherence, on the occurrence of DRP in elderly patients of 60 year and older discharged from the hospital using five or more drugs. The hypothesis of this study is that this multifaceted intervention will reduce the occurrence of DRP and improve compliance. The secondary objectives are to evaluate the effect of the intervention on: attitude to drugs, incidence of Re-hospitalisations related to medicines, functional status of the patient, quality of life. Moreover, the cost-effectiveness of this intervention will be analysed.

## Methods/Design

### Design of the study

**Figure 2:**
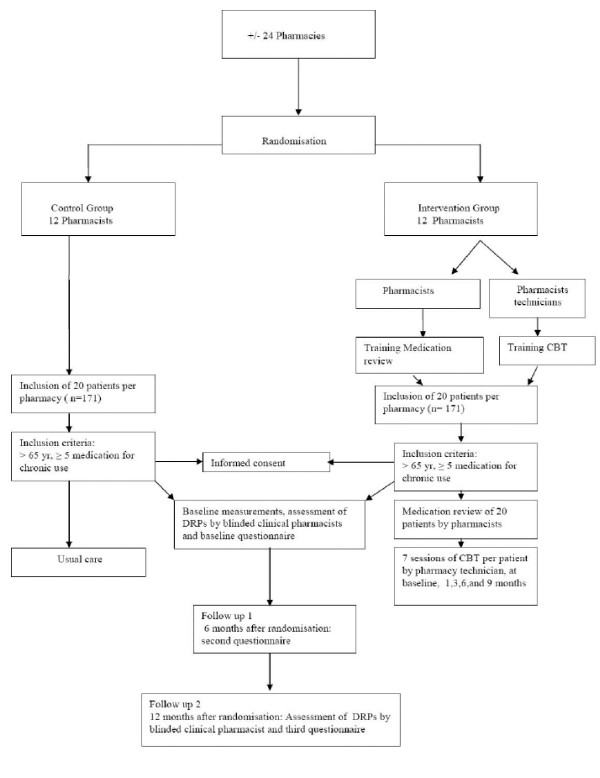
**Study Design**.

The study is designed as a randomized, controlled, intervention study involving 342 patients with a follow-up of one year. The intervention will be performed by community pharmacists. A flow-chart of the study is shown in Figure 2.

The Medical Ethics Committee of the VU University medical enter in Amsterdam approved the study design, protocols, information letters and informed consent form.

### Setting

The trial will be conducted in 23 community pharmacists in Amsterdam, Amstelveen, Hoofddorp and Diemen, the Netherlands. The medication review will be performed by a pharmacist in close collaboration with the general practitioner (GP). The patients will be counseled at home or in the pharmacy by a pharmacy technician.

### Study Population

A total of 342 patients discharged from any department, except oncology and psychiatry, from any general and academic hospital in Amsterdam, Amstelveen, Hoofddorp and Diemen will be enrolled into the study. Patients 60 years and older, using five or more prescription-only chronic drugs when admitted to the hospital are considered eligible to participate into the study. The pharmacists will evaluate whether the patient uses 5 or more chronic drugs. Furthermore, to participate, the patients have to give written informed consent.

### Treatment allocation

Ten Pharmacotherapeutic Audit Meetings (PTAMs) will participate in the study. In the Netherlands general practitioners and pharmacists are organized in PTAMs. Within a PTAM pharmacists are randomized as a control or intervention pharmacist.

### Study procedures

#### Intervention group

##### Medication review

The medication of patients in the intervention group will be reviewed by the community pharmacist using the full record of prescription only drugs which were dispensed by the patients' pharmacy and the patients medication evaluation profile. This profile shows when the patients has obtained his medication from the pharmacy. The GP will be consulted by telephone for details about indications for drugs and results from laboratory tests. When prescribed by a medical specialist, details about the indication for the drug will be obtained from the specialist, who will be consulted by the community pharmacist. The National guidelines for treatment of diseases will be used by community pharmacists as a method for performing medication review.

The Pharmaceutical care network Europe DRP-score form will be used to record drug problems. Each drug will be evaluated on adverse reactions, drug choice problems, dosing problems, drug use problems, drug-interactions or other problems. Causes for drug related problems will be assessed and interventions will be made. During the medication review the patient will be involved as a full partner. Any changes made will be communicated with the patient. This method for medication review will be pilot tested before use.

The occurrence of drug related problems will be discussed with the GP. The result may be an adaptation of the drug regimen. The medication review will take 10-30 minutes per patient and will depend on the complexity of the medication regimen and problems detected.

##### Cognitive behaviour treatment

Patients randomised to the intervention group will also receive cognitive behaviour treatment (CBT) at baseline and 1, 3, 6, 9 and 12 months by a pharmacy technician, with help of a structured interview protocol and with use of communication and motivational interviewing skills at home or in the pharmacy. The first session will be within one week of inclusion in order not to delay participant program admission. During these sessions the result of the medication review will be discussed with the patient. The patient will be informed about the effects, side effects and use of the drugs. Patients will be counselled according to the motivational interviewing principle to sustain or improve their drug adherence. The patients understanding of his or hers condition and its treatment are considered when appropriate. If possible, home supplies of drugs are checked and rationalised at each visit. All patients receive a written outline of their drug regimen. Cancelled and redundant drugs are taken in. During the session, over the counter remedies will be included in the medication review. All sessions are done by pharmacist technicians with help of a structured protocol. The patient visit will take 30-60 minutes.

##### Training course pharmacists

All participating intervention pharmacists will participate in a one day medication review training course. During this training session the background of medication review will be explained. The use of Medication Evaluation Profiles will be explained. Furthermore, the way the PCNE DRP-score form should be used will be explained.

To pilot test the interrater variability of the PCNE DRP-score form and the efficacy of the medication review training all participating pharmacists (controls and intervention pharmacists) will receive 5 medication overviews of test-patients with additional information about clinical notes approximately three months before the medication review training session. The results of the medication reviews will be evaluated by a specialist in medication review.

When intervention pharmacists have completed the training course in medication review, all pharmacists (control and interventions) will be asked to review five new test-patients. The intervention pharmacists are expected to detect more DRPs afterwards than control pharmacists who did not receive a special training in medication review. The results of the medication reviews will be evaluated by the same specialist as before.

##### Training pharmacy technicians

All intervention technicians will participate in a two days training course of motivational interviewing and a one day course of problem solving treatment. During these training sessions pharmacy technicians will be lectured about communication skills such as Motivational interviewing and Problem solving treatment skills by a specialized psychologist on this subject. In addition pharmacy technicians will receive coaching on the job. Video tapes of interviews with patients will then be recorded and reviewed by two independent researchers using the Motivational Interviewing Treatment Integrity Code score method to evaluate the quality of the motivational interviewing skills of the pharmacy technicians.

##### Pharmacotherapeutic Audit Meetings (PTAM)

In addition, to improve the intervention and its performance, the intervention will be discussed in Pharmacotherapeutic Audit Meetings (PTAM). At PTAM meetings agreements are made to improve the pharmacotherapeutic care provided. A PTAM-group generally consists of 2 to 6 pharmacists from 2 to 3 pharmacies and 6 to 10 general practitioners. During this study three PTAM sessions are organized. The first to discuss the intervention. The second to make the agreements and the third PTAM sessions are used to evaluate, the procedure.

#### Control Group

Patients of control pharmacists will receive usual care according to the Dutch Pharmacy Standard (Table 1). Furthermore, patients are visited once by a pharmacy technician and asked about possible drug related problems. These problems will be registered for scientific use only.

**Table 1:** Usual care, according to the Dutch Pharmacy Standard

1	Preceding the release check, prescriptions are routinely checked for drug interactions and contra-indications by the PAIS.
2	Discharge medication is delivered at the patients' home or is picked up by the patient or carer in the pharmacy.
3	Drugs are routinely delivered with a drug information leaflet but patients often are also handed supplementary personalized PAIS-generated information letters on newly prescribed drugs.
4	When the discharge medication is collected from the pharmacy, the patient or carer is also provided with additional oral information about newly prescribed drugs. This includes an explanation of the drugs' actions, their use and of possible side effects.

First edition of 1996. The current (2nd) edition was introduced in 2006 (KNMP, The Hague).

### Outcome Measures

#### Primary outcome measures

Using a checklist including common drug problems, the prevalence of DRP in patients of control and intervention pharmacists will be determined at base-line and at 12 months by a team of independent clinical pharmacologists. The identification of drug problems by community pharmacist will be assessed on the basis of a structured interview of the patient and the medication overview of the patient registered in the pharmacy information and administration system.

#### Adherence with drug treatment

The 5-item self-report Medication Adherence Rating Scale (MARS) will be used to assess medication adherence [24].

#### Secondary outcome measures

##### Incidence of Re-hospitalization

The total incidence of hospital re-admissions possibly related to medicine during the study period is measured. The incidence of hospital admissions will be derived from general practitioners medical record and determined by self report. The relation to drugs will be derived using a list of selection criteria. Hospital admissions will be classified as related to drugs if:

▪ It is explicitly clarified in the medical notes that the admission is drug related. The specialist of the patient will be consulted to clarify the relation to drugs.

▪ The patients' physician, when asked, indicates that the admission was drug related.

▪ To classify the causality of the hospital admission to the drug, the Naranjo algorithm will be used. The Naranjo algorithm or Naranjo Scale is a questionnaire designed by Naranjo *et al *for determining the likelihood of whether an ADR is actually due to the drug rather than the result of other factors [25]. The causality will be determined by the project leader.

All participating patients are asked to complete 3 validated questionnaires during the research year. The first questionnaire will be sent to the patients one week before the first counselling visit of the pharmacist. The second questionnaire will be sent at 6 months, the third questionnaire will be sent at 12 months. It will take approximately 30 minutes to complete one questionnaire.

### Beliefs about Medicines will be assessed by questionnaire

- Beliefs about Medicines Questionnaire (BMQ) [26,27].

- Attitude towards prescribed medicine [28].

### Functional status of the patient

Functional status of the patient will be determined by examining the following dimensions: physical functioning, role limitation due to physical health problems, bodily pain, general health perceptions, vitality, social functioning and general mental health. These dimensions will be determined using the RAND-36 questionnaire [29].

### Patient satisfaction about prescribed medicines

Patients satisfaction will be determined using the Satisfaction with Information about Medicines questionnaire (SIMS) [30].

### Quality of life

Quality of life: mobility, self- care, main activity (work, study, housework, leisure), social relationships, pain, mood will be assessed using the EuroQol [31].

### Costs effectiveness

Costs will be registered by the pharmacists in terms of time and material spent on the counselling of patients. Health care costs made by the patient will be assessed from a societal perspective using monthly costs calendars in which direct and indirect costs will be prospectively determined by the patient. The following costs will be included in the calendars.

▪ Visits to a General Practitioner (including home visits)

▪ Visits to a medical specialist

▪ Visits to a physiotherapist

▪ Hospitalisations or admission to a nursing home

▪ Informal care (from neighbours, family or friends)

▪ Alternative medical treatment.

Costs of prescription-only medication use will be derived from the pharmacy system.

### Sample size

The primary outcome measure is the result of the medication review and patient counselling on the occurrence of DRPs. Based on previous studies it is estimated that the percentage of DRP in the patients of control pharmacists is 30%. It is estimated that the intervention reduces the number of preventable DRP with one third (33%). With a type 1 error of 0.05, a power of 80%, and a ration of one between both groups of patients, multilevel randomisation resulting in a loss of power of 15%, a total of 342 patients is needed to show a statistically significant difference. The estimated number of patients fulfilling the criteria and willing to participate is 4 per pharmacy per month. With an actual recruitment time of 6 months, and about 20 participating pharmacies, the number of 342 patients will be achieved.

### Analyses

Descriptive statistics (means ± SD or median and interquartile range in case of a skewed distribution) will be used to describe the whole study sample with regard to demographics, drug use and reason for previous hospital admission. The analyses will be conducted according to the intention-to-treat principle. To assess the impact of the intervention, multilevel linear and logistic regression analyses will be conducted to study differences in outcome measures between patients of the intervention and control pharmacies. Using multilevel analyses enables taking clustering of observations of participants receiving care from the same GP and for repeated measurements within one subject in account. Differences in changes between groups are measured with 95% confidence intervals. We will adjust data for possible confounders and effect modifiers (age, sex, ethnicity, level of education). Separate analyses of effect modifiers and mediators will be conducted in order to gain a better understanding which subgroups benefit most from the intervention. Confidence intervals of differences in costs between groups will be estimated using bootstrapping methods. (Bootstrapping statistics is a general method for performing statistical analyses without making strong parametric assumptions [32].

## Discussion

This article presents a description of a RCT, which aims to investigate whether a multifaceted intervention by the community pharmacist existing of medication review and CBT to increase drug adherence after discharge from the hospital, is effective in reducing DRPs among elderly patients of 60 years and older using five or more prescript drugs for a chronic disease. The present paper will provide other researchers working on this subject, GPs, pharmacists, medical specialists, other health care providers and policy makers an overview of the RCT. This enables them to critically review the methodological quality, the background theory and the practical issues of the RCT. Previous studies have shown that pharmacist initiated interventions are effective in reducing DRPs [33,34]. To our knowledge this is the first study that will combine two strategies: medication review and CBT to reduce the occurrence of DRPs among patients who are at higher risk to these problems. Studies have shown that medication review is an effective method in decreasing the occurrence of DRPs [34]. Most studies have studied the effect of medication review on patients in all age categories on the occurrence of drug problems. In this study we will determine the effect of medication review on the occurrence of DRPs among elderly patients who are discharged from the hospital. This has not yet been studied before. Furthermore, we expect that patient CBT based on motivational interviewing and problem solving treatment will increase or sustain patient's drug adherence after hospital discharge and therefore lowers the risk of having a DRPs and re-hospitalisation.

There are several practical limitations associated with performing medication review. Detecting and solving drug related problems by health care workers such as community pharmacists is a time consuming process [35]. In order to make any changes in the drug regimen, the pharmacist has to contact the general practitioner first, which is time consuming. Furthermore, community pharmacists have to communicate the changes that are made with their patients. Because of their busy daily schedules, it may be possible that pharmacists do not find that medication review has a priority. Another major limitation is the possible bias in the detection and solving of DRPs by the different community pharmacists since all pharmacists have different experiences in performing a medication review. Some pharmacists will therefore identify more or less drug related problems than others, depending on the limited time. In addition, another limitation of the intervention is that the performance of CBT might differ between pharmacy technicians because of the interview skills they have. It is therefore difficult to decide whether all patients receive the same therapy sessions. We will try to reduce the differences between the pharmacy technicians, by using a structured interview protocol and motivational interviewing and problem solving treatment courses. A major strength is by combining medication review and CBT sessions can lead to a possible improvement of detection of drug related problems. The CBT sessions with patients discharged from the hospital will be helpful in detecting drug problems such as side effects and non-compliance, and thus lead to a better communication between the community pharmacy and patient.

A limitation of the study design is that there is no literature available that describes an evidence based method that can be used by pharmacists to report the quality of the pharmacotherapy or how to detect and solve drug related problems. However, we will apply in this study a method of prescription problems which we can test on feasibility and effectiveness. Strength of this study design is that it is a RCT.

The inclusion of the patients has been performed between in January 2008 and March 2009. Duration of the study will be one year. If this study has a positive effect in reducing the occurrence of drug related problems, it can be implemented as within a community pharmacy. This service can be specially implemented for patients discharged from the hospital, in order to minimize the occurrence of DRPs.

## Competing interests

The authors declare that they have no competing interests.

## Authors' contributions

AA is responsible for the data collection and wrote the manuscript. JH, GN developed the original idea for the study. The study design was further developed by JH, AA, LW, JD and GN. All authors have read and approved the final manuscript.

## Pre-publication history

The pre-publication history for this paper can be accessed here:

http://www.biomedcentral.com/1471-2458/10/133/prepub
